# TRIM14 regulates melanoma malignancy via PTEN/PI3K/AKT and STAT3 pathways

**DOI:** 10.18632/aging.203003

**Published:** 2021-05-11

**Authors:** Jiangyan Chen, Lin Huang, Jin Quan, Debing Xiang

**Affiliations:** 1 Department of Oncology, Jiangjin Central Hospital of Chongqing, Chongqing, China; 2 Department of Dermatology, Jiangjin Central Hospital of Chongqing, Chongqing, China

**Keywords:** TRIM14, melanoma, AKT, STAT3

## Abstract

Melanoma is one of the most aggressive cancers with poor overall survival. To date, there are still few effective methods for the treatment of melanoma. TRIM14 was previously reported to be an important oncogene in many tumors. Nevertheless, the roles of TRIM14 in melanoma remain unknown. In this study, we found that TRIM14 was abnormally upregulated in melanoma cell lines. Knockdown of TRIM14 suppressed melanoma cell proliferation, migration, invasion, epithelial-mesenchymal transition, and melanin synthesis. Overexpression of TRIM14 had opposite effects on the cellular functions of melanoma cell lines. Further study revealed that TRIM14 knockdown increased PTEN protein levels, which in turn inactivated AKT and STAT3 pathways. Moreover, blocking AKT or STAT3 pathway with a specific inhibitor could partially reverse the promotion of melanoma malignancy mediated by TRIM14 overexpression. In addition, *in vivo* assay also supported the above findings. These results indicated that TRIM14 might be a promising target for melanoma treatment.

## INTRODUCTION

Melanoma is one of the most aggressive diseases with poor overall survival [1]. According to statistics, there are over 73,000 cases of melanoma newly diagnosed per year with an overall upward trend in the United States [2]. Despite aggressive treatments like surgery, chemotherapy, and radiotherapy, there is no obvious impact on improving the overall survival of patients with melanoma [3]. In recent years, targeted therapy and immunotherapy have been proved to improve the prognosis of patients with melanoma, especially metastatic melanoma [4, 5].

TRIM proteins represent an ancient protein family, including at least 77 TRIM proteins in humans. TRIM family, as one of the largest subfamilies of ubiquitin E3 ligases, has been reported to govern tumor development and progression [6, 7]. In colorectal cancer, TRIM23 acts as an oncogene and contributes to carcinogenesis [8]. In glioma, TRIM21 promotes cell growth, cell invasion, and suppresses cellular senescence [9]. In addition, TRIM25 targets the Keap1-Nrf2 pathway to regulate cell proliferation and survival of hepatocellular carcinoma [10]. Furthermore, TRIM4, TRIM16, TRIM17, TRIM28, and TRIM31 have also been shown to play crucial roles in melanoma [11–14]. Thus, to deeply explore the roles of the TRIM family is meaningful for the targeted therapy of melanoma.

TRIM14, one member of the TRIM family, has been proven to function as an important oncogene. Xu et al. reported that TRIM14 was aberrantly upregulated in human osteosarcoma tissues and cell lines. High TRIM14 expression generally means a poor survival of patients. Overexpression of TRIM14 promoted tumor cells proliferation and invasion through the AKT pathway [15]. In colon cancer, TRIM14 promoted cancer cell invasion by targeting the SPHK1/STAT3 pathway [16]. In breast cancer, TRIM14 enhanced tumor cell proliferation via inhibiting apoptosis [17]. Moreover, TRIM14 also participated in regulating glioma chemoresistance and epithelial-mesenchymal transition [18, 19]. Nevertheless, the roles of TRIM14 in melanoma remain unknown.

In the present study, we found that TRIM14 is upregulated in melanoma cell lines compared with normal cells. Knockdown of TRIM14 inhibits tumor cell proliferation, clone formation, invasion, cycle arrest, and melanin content of melanoma cells. The overexpression of TRIM14 does the opposite function. In addition, PTEN/PI3K/AKT and STAT3 pathways participate in regulating TRIM14-induced melanoma development and progression. Finally, tumor formation in nude mice also showed that TRIM14 could regulate melanoma tumor growth.

## MATERIALS AND METHODS

### Cell culture and transfection

The human epidermal melanocytes (HEMa-LP) and melanoma cell lines (A375, SK-MEL-24, WM451, WM35) were purchased from the American Type Culture Collection (Manassas, VA, USA) and incubated with DMEM medium containing 10% fetal bovine serum in an incubator at 37°C. When the cell confluence was over 80%, melanoma cells were seeded in 6-well plates and cultivated for 24 h before transfection. After that, melanoma cells were transfected with TRIM14 knockdown (siRNA-TRIM14) or overexpression (Oe-TRIM14) plasmid and their corresponding negative controls (siRNA-NC or Oe-TRIM14) for the indicated time, and then they were used for the following assays.

### CCK-8 assay

For cell viability assay, 2000 pretreated cells were added in duplicate in 96-well plates and incubated for 24 h at 37°C with 5% CO_2_. Then, the proliferation of melanoma cell was detected at 24, 48, and 72 h with Cell Counting Kit-8 according to the manufacturer’s protocol respectively.

### Clone formation assay

The pretreated cells were seeded into 6-well plates at a density of 800 cells/well and then cultured for 15 days. The culture medium was changed five times. Fifteen days later, the cells were washed with PBS three times, and fixed with 4% paraformaldehyde for 10 min and stained with 0.1% crystal violet dye for 10 min. Finally, clone formation of melanoma cells was counted and photographed under an inverted microscope.

### Wound healing assay

The pretreated cells were seeded into 6-well plates in duplicate and cultured in DMEM without fetal bovine serum until it was about 100% confluence. 10 μl pipette tips were used to scratch cell monolayer in a straight line and then cells were washed with PBS three times. Cells were cultured in DMEM without fetal bovine serum for 48 h and the wound width was imaged to detect the capacity of tumor cell migration.

### Transwell invasion assay

For invasion assay, the pretreated cells were resuspended in serum-free DMEM, and a cell suspension containing 5 × 10^4^ pretreated cells (total volume 200 μL) were seeded on the upper chamber coated with Matrigel and 600 μL DMEM medium with 10% serum was added to the lower chamber. Cells were incubated using 24-well transwell plates for 48 h. Then cells were in sequence fixed in 4% paraformaldehyde and stained by crystal violet for a certain time. Finally, the number of the invasive cells was counted under a microscope.

### Real-time PCR

Total RNA was extracted from treated melanoma cells by Trizol buffer according to the manufacturer's instructions. According to the manufacturer’s protocol of the Quantitative Reverse Transcription Kit and SYBR Green PCR Kit, the mRNA levels of related genes were evaluated by RT-qPCR. The relative quantification of each gene expression was calculated via normalization against GAPDH using the 2^-ΔΔCT^ method.

### Western blot assay

Total proteins of pretreated melanoma cells were extracted by Radio-Immunoprecipitation Assay buffer. 50 ng proteins of each sample were injected into a Bis-Tris SDS/PAGE gel and transferred to PVDF membranes. The membranes were blocked with 5% BSA for 1 h at room temperature and then incubated with related primary antibodies overnight. After incubating with corresponding secondary antibodies and ECL kit, bands were then analyzed with an imaging system.

### Melanin content of melanoma cells assay

The pretreated melanoma cells were added to six-well culture plates and incubated for 24 h. After that, cells were harvested and washed three times with PBS buffer (pH 7.2). Then, the above cells were lysed in 500 μl of 1 M NaOH at 90°C for 1 h. Finally, the lysates were centrifuged at 3000 g for 10 min and the absorbance was measured at 405 nm.

### Flow cytometry analysis

After 48 h of incubation, melanoma cells were collected and fixed in ice-cold 70% ethanol overnight. After washing with PBS, cells were incubated with 1 mg/ml RNase A at 37°C for 30 min, and stained with propidium iodide for 30 min in the dark. Cell cycle distribution was analyzed by flow cytometry with CellQuest software (Becton Dickinson).

### Xenograft tumor model in nude mice

Six-week-old male BALB/c nude mice were used in this assay. The nude mice were assigned to the following several groups: control; si-TRIM14; Oe-TRIM14; Oe-TRIM14+LY2940 and Oe-TRIM14+SH-4-54. A375 melanoma cells were transfected with si-TRIM14 or Oe-TRIM14 plasmid with or without pretreatment of LY2940/SH-4-54. After that, these cells were made into a cell suspension and subcutaneously injected into the marked mouse. The tumor size was recorded and the tumor volume was calculated as follows: volume = 1/2 × length × width^2^. The tumor was peeled off and weighed on day 20 to weigh and take pictures, and it was then used to detect related protein expression using western blot.

### Statistical analysis

All the experimental data are expressed as the means ± SD. SPSS 22.0 software (IBM Corp.) was used for data analyses. Statistic comparison was performed using the one-way ANOVA analysis. When a *P* was <0.05, it was regarded as statistically significant.

## RESULTS

### TRIM14 expression was upregulated in melanoma cells

**Figure 1:**
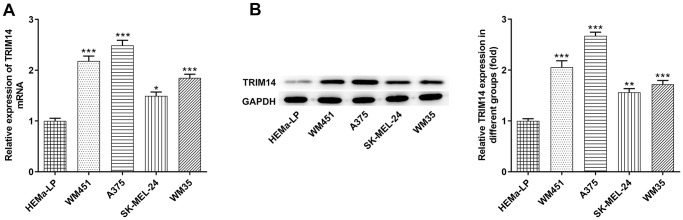
**TRIM14 was upregulated in melanoma cell lines.** The human epidermal melanocytes (HEMa-LP) and melanoma cell lines (A375, SK-MEL-24, WM451, WM35) were used to detect the mRNA level of TRIM14 using RT-qPCR (**A**) and the protein expression of TRIM14 using western blot (**B**). ^*^, ^**^, ^***^p < 0.05, 0.01, 0.001 vs HEMa-LP.

To explore the expression of TRIM14 in melanoma cells, RT-qPCR and Western blot assays were performed. As shown in Figure 1A, mRNA expression of TRIM14 in melanoma cell lines (A375, SK-MEL-24, WM451, WM35) was higher than that in HEMa-LP cells. Further western blot assay presented a similar result, displaying that TRIM14 was up-regulated in melanoma cells (Figure 1B). Furthermore, the expression of TRIM14 in melanoma WM451 and A375 cell lines was the highest. Thus, WM451 and A375 cell lines were used in the subsequent knockdown experiments.

### Knockdown of TRIM14 suppressed melanoma cell proliferation

**Figure 2:**
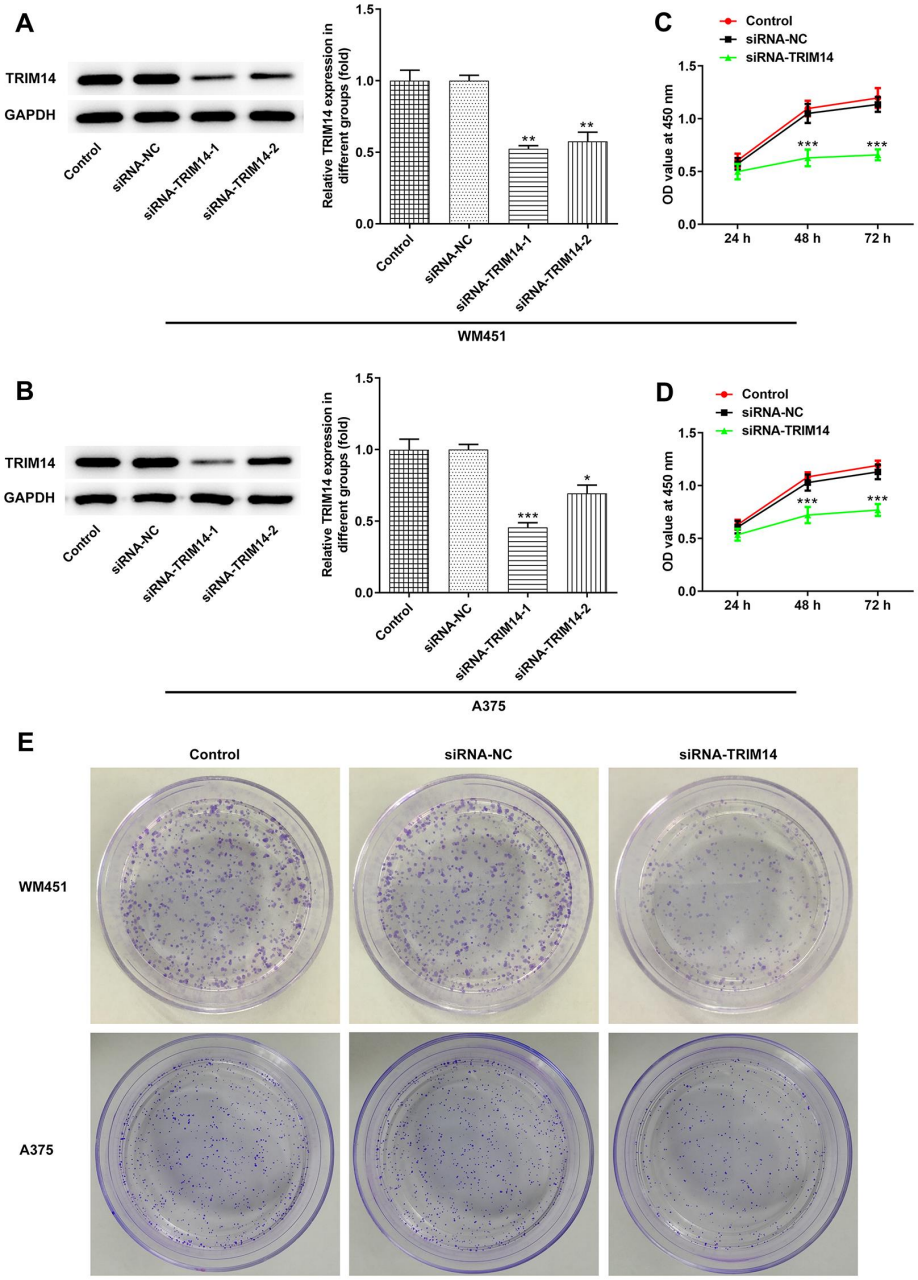
**Knockdown of TRIM14 inhibited melanoma cell proliferation.** Both WM451 and A375 cells were transfected with siRNA-NC and siRNA-TRIM14-1/2, then the protein expression of TRIM14 in each group was measured using western blot (**A**–**B**). After transfection, the cell proliferation was determined using CCK-8 assay (**C**–**D**) and clone formation assay (**E**). ^*^, ^**^, ^***^p < 0.05, 0.01, 0.001 vs siRNA-NC.

First, we constructed the interference plasmid of TRIM14 and detected its interference effects. Compared with control or negative control, levels of TRIM14 expression were lower in TRIM14-1 knockout and TRIM14-2 knockout group. What’s more, the expression of TRIM14 in TRIM14-1 knockout group was lower than TRIM14-2 knockout group (Figure 2A, 2B). Therefore, TRIM14-1 interference plasmid was selected for subsequent assays. As shown in Figure 2C–2E, proliferation and clone formation capacity of WM451 and A375 cell lines were significantly decreased in the TRIM14 knockdown group compared with control or negative control. These results demonstrated that TRIM14 knockdown inhibited melanoma cell proliferation and clone formation.

### Knockdown of TRIM14 inhibited melanoma cell migration and invasion

**Figure 3:**
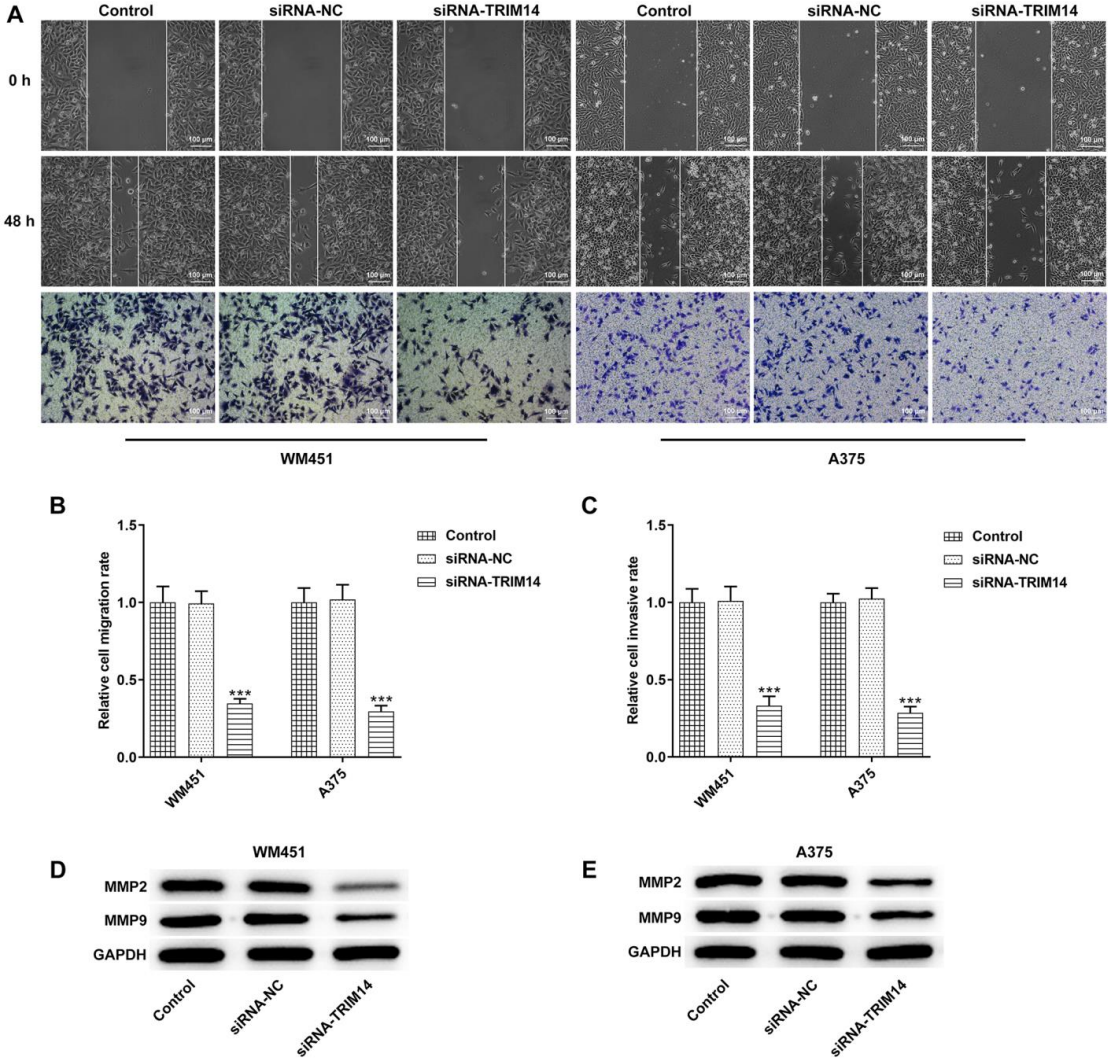
**TRIM14 knockdown suppressed melanoma cell migration and invasion.** After transfection in both WM451 and A375 cells, wound-healing assay and Transwell assay were applied to determine cell migration and invasion abilities, respectively (**A**–**C**). The protein expression of MMP2 and MMP9 in different groups of WM451 and A375 cells were measured using western blot (**D**–**E**). Magnification ×100. ^***^p < 0.001 vs siRNA-NC.

To investigate the roles of TRIM14 in melanoma cells migration and invasion, wound healing and transwell assays were performed. Following TRIM14 silencing, the scratch area did not reduce and number of invasive cells decreased in comparison to control or negative control (Figure 3A–3C). In addition, Matrix Metalloproteinase-2 (MMP-2) and MMP-9 were key proteins of cell invasion, and knockdown of TRIM14 also reduced levels of MMP-2 and MMP-9 proteins (Figure 3D, 3E). These results revealed that TRIM14 knockdown suppressed melanoma cells’ migration and invasion.

### Inhibition of TRIM14 induced melanoma cell cycle arrest

**Figure 4:**
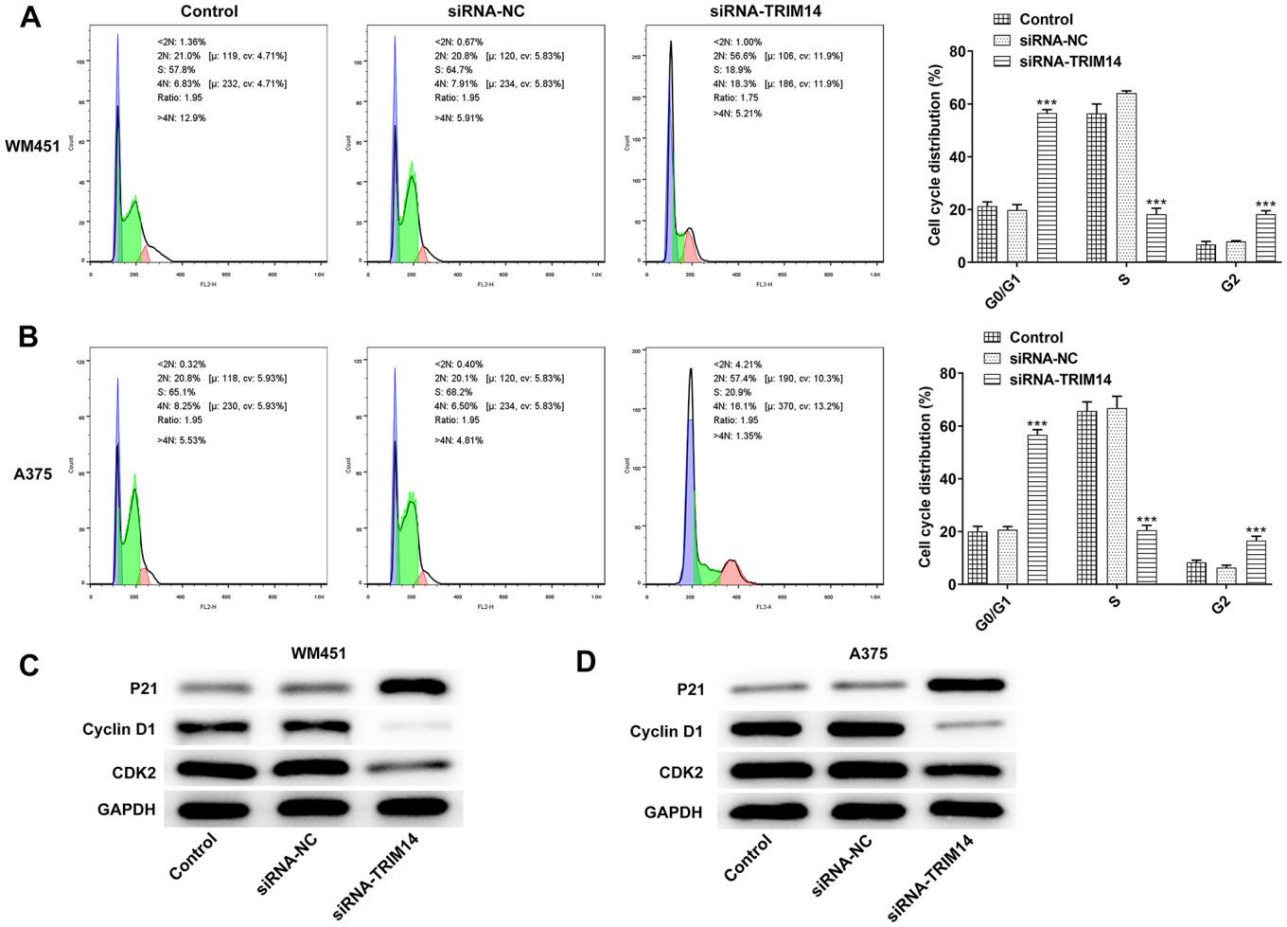
**Knockdown of TRIM14 induced melanoma cell cycle arrest.** After transfection, both WM451 and A375 cells were collected for cell cycle distribution analysis using flow cytometry assay (**A**–**B**). The expression of cell cycle-related proteins (p21, cyclin D1, CDK2) were detected using western blot (**C**–**D**). ^***^p < 0.001 vs siRNA-NC.

To further validate the biological function of TRIM14 in melanoma cell cycle, flow cytometry was used to detect cell cycle. As shown in Figure 4A, 4B, knockdown of TRIM14 significantly increased cell population in G0/G1 phase, and decreased the cell population in S phase, indicating that knockdown of TRIM14 promoted cell cycle arrest in G1 phase. Knockdown of TRIM14 also decreased the expression of cyclin-dependent kinase 2 (CDK2) and cyclin D1 proteins while increased the expression of Cyclin-dependent kinase inhibitor p21 in both WM451 and A375 cells (Figure 4C, 4D). Thus, the above results suggested that TRIM14 knockdown could regulate cell cycle distribution by promoting cell cycle arrest in G1 phase.

### TRIM14 knockdown inhibited melanin synthesis of melanoma cells

**Figure 5:**
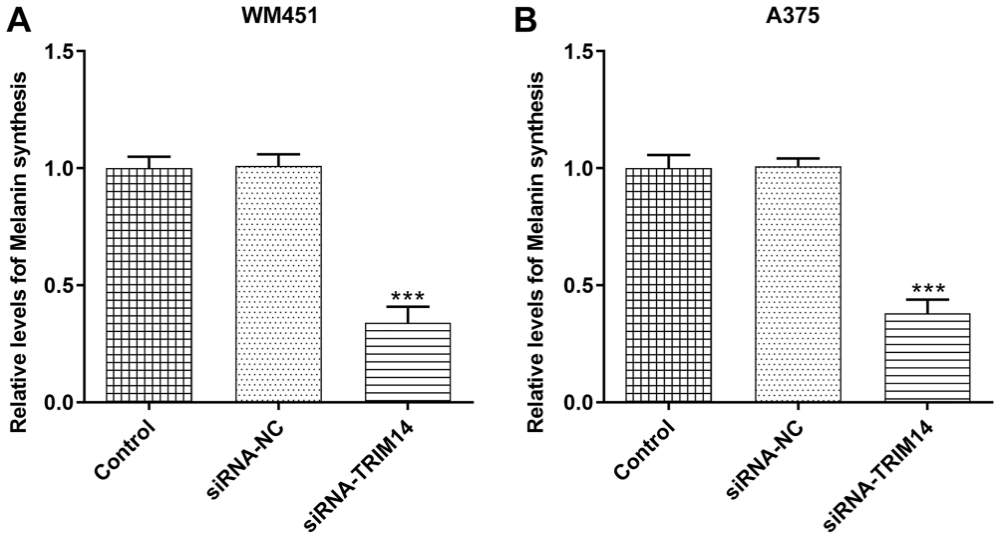
**TRIM14 knockdown downregulated melanin synthesis of melanoma cell.** After transfection, the melanin synthesis abilities of different groups in both WM451 and A375 cells were measured (**A**–**B**). ^***^p < 0.001 vs siRNA-NC.

To our delight, TRIM14 also had a role in regulating melanin synthesis in melanoma cells. As shown in Figure 5A, 5B, compared with control or negative control, knockdown of TRIM14 suppressed melanin synthesis of WM451 and A375 cells. This result indicated that the melanin synthesis of melanoma cells was also regulated by TRIM14.

### Knockdown of TRIM14 inhibited PI3K/AKT and STAT3 pathways activation

**Figure 6:**
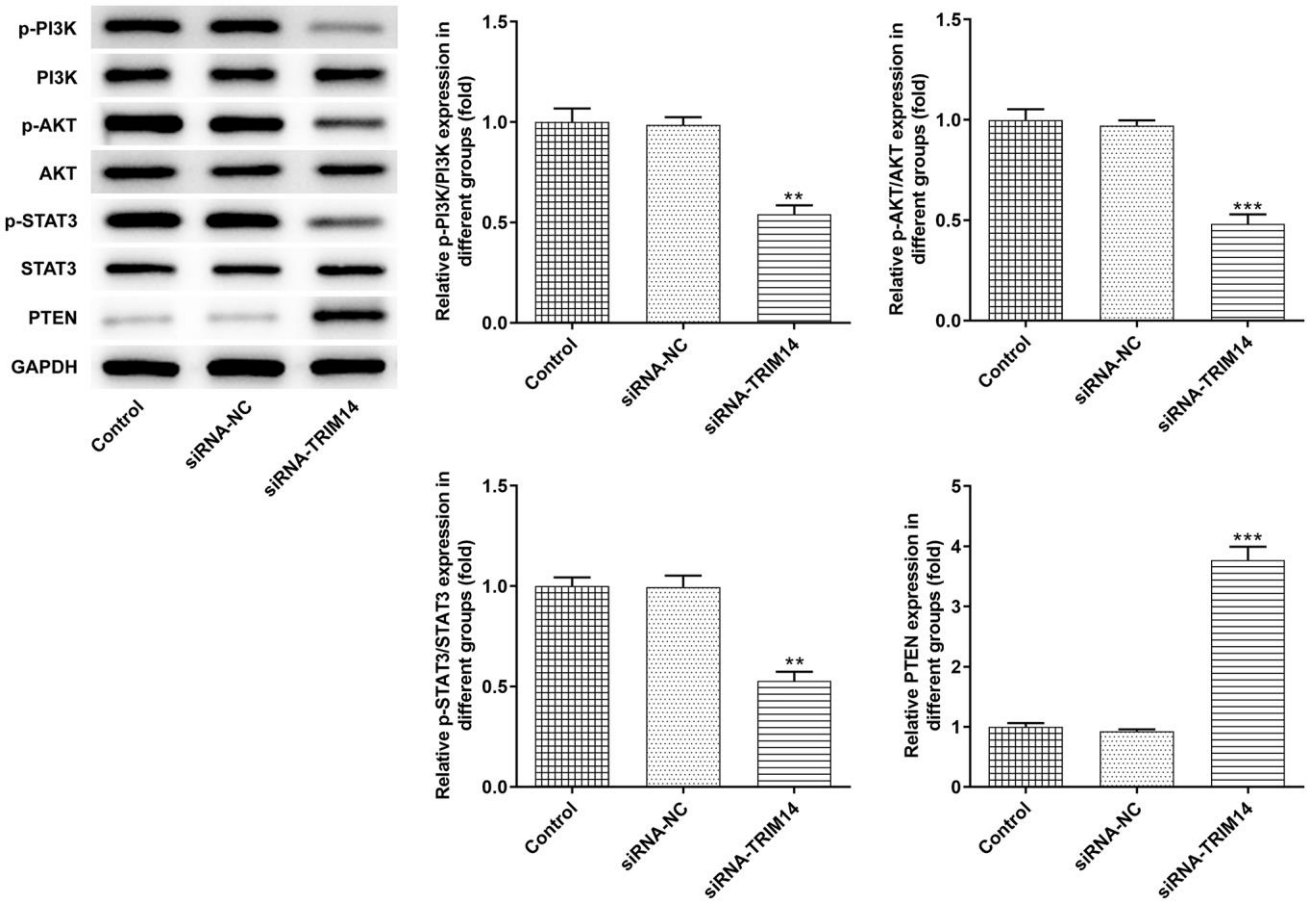
**Knockdown of TRIM14 inactivated AKT and STAT3 pathways.** A375 cells were transfected with siRNA-NC and siRNA-TRIM14, respectively. The protein expression of PTEN, p-PI3K/PI3K, p-AKT/AKT, p-STAT3/STAT3 was detected using western blot. ^**^, ^***^p < 0.01, 0.001 vs siRNA-NC.

Previous studies reported that TRIM14 colocalizes PTEN in the cytoplasm and induces PTEN ubiquitination to suppress activation of PI3K/AKT and STAT3 pathways [15, 16, 20]. In A375 cells, we silenced TRIM14 and detected related protein expression. As shown in Figure 6, inhibition of TRIM14 increased levels of PTEN protein. We noticed that TRIM14 had no evident impact on total PI3K, AKT, and STAT3 protein expression. However, phosphorylation of PI3K, AKT, and STAT3 protein expression was decreased following TRIM14 knockdown. These results demonstrated that TRIM14 could regulate PI3K/AKT and STAT3 pathways in melanoma cells.

### Overexpression of TRIM14 promoted melanoma cell proliferation through AKT and STAT3 pathways

**Figure 7:**
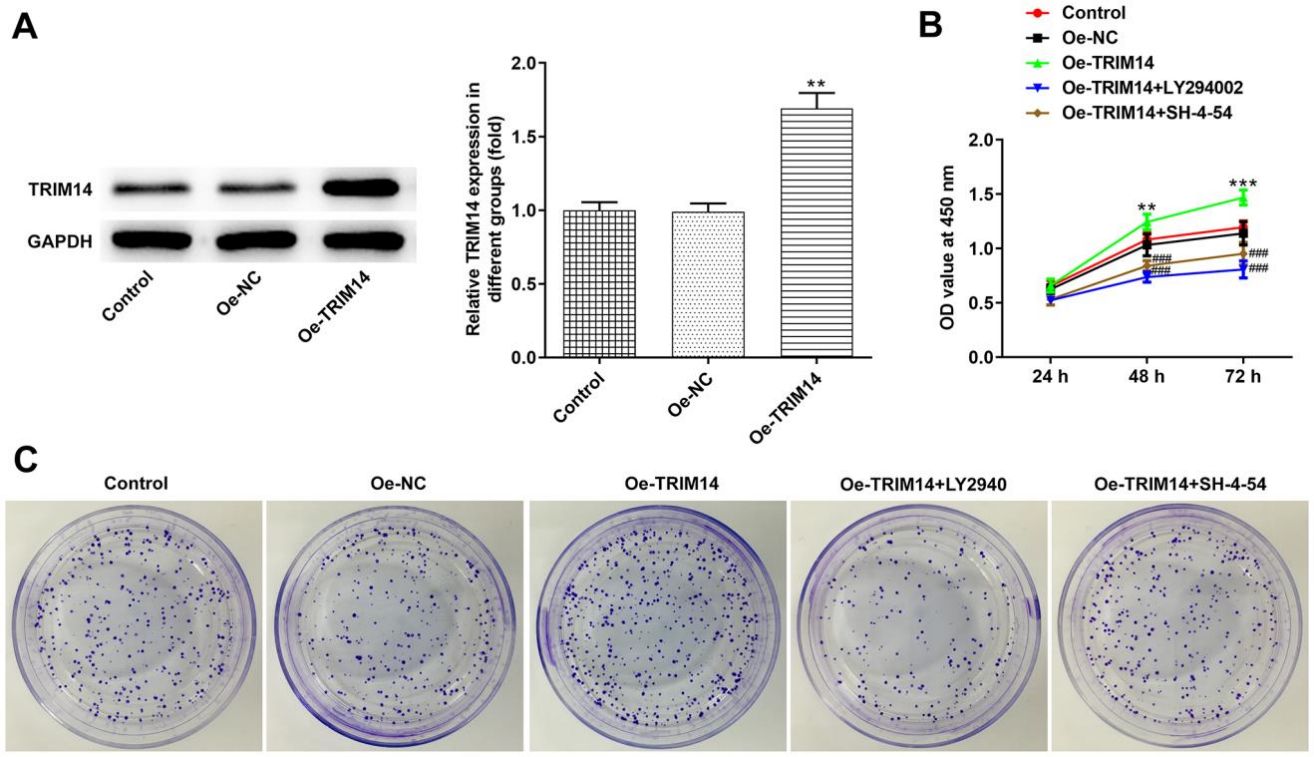
**Overexpression of TRIM14 promoted melanoma cell proliferation via AKT and STAT3 pathways.** A375 cells were transfected with Oe-NC and Oe-TRIM14, and the protein expression of TRIM14 after transfection was determined using western blot (**A**). A375 cells were transfected with Oe-TRIM14 with or without the treatment of LY294002 (an AKT pathway inhibitor) or SH-4-54 (a STAT3 pathway inhibitor). After transfection, cell proliferation ability was determined using CCK-8 assay (**B**) and clone formation assay (**C**). ^**^p < 0.01 vs Oe-NC; ^###^*p* < 0.001 vs Oe-TRIM14.

Considering knockdown or overexpression of TRIM14 might play different roles in melanoma cells, we then constructed the TRIM14 overexpression plasmid. As shown in Figure 1, the expression of TRIM14 in A375 cells was the lowest among melanoma cell lines, and thus the A375 cell line was chosen for overexpression experiments. We could observe that the TRIM14 overexpression plasmid was constructed successfully (Figure 7A). After that, melanoma cells were pretreated with LY294002, an AKT pathway inhibitor, or SH-4-54, a STAT3 pathway inhibitor, and then transfected with TRIM14 overexpression plasmid. As shown in Figure 7B, 7C, overexpression of TRIM14 significantly promoted growth and clone formation capacity of melanoma cells, while blocking AKT or STAT3 pathway partially reversed the promotion of melanoma cell proliferation mediated by TRIM14. These results revealed that overexpression of TRIM14 could activate AKT and STAT3 pathways to regulate melanoma A375 cell proliferation.

### TRIM14 overexpression enhanced melanoma cell migration and invasion via AKT and STAT3 pathways

**Figure 8:**
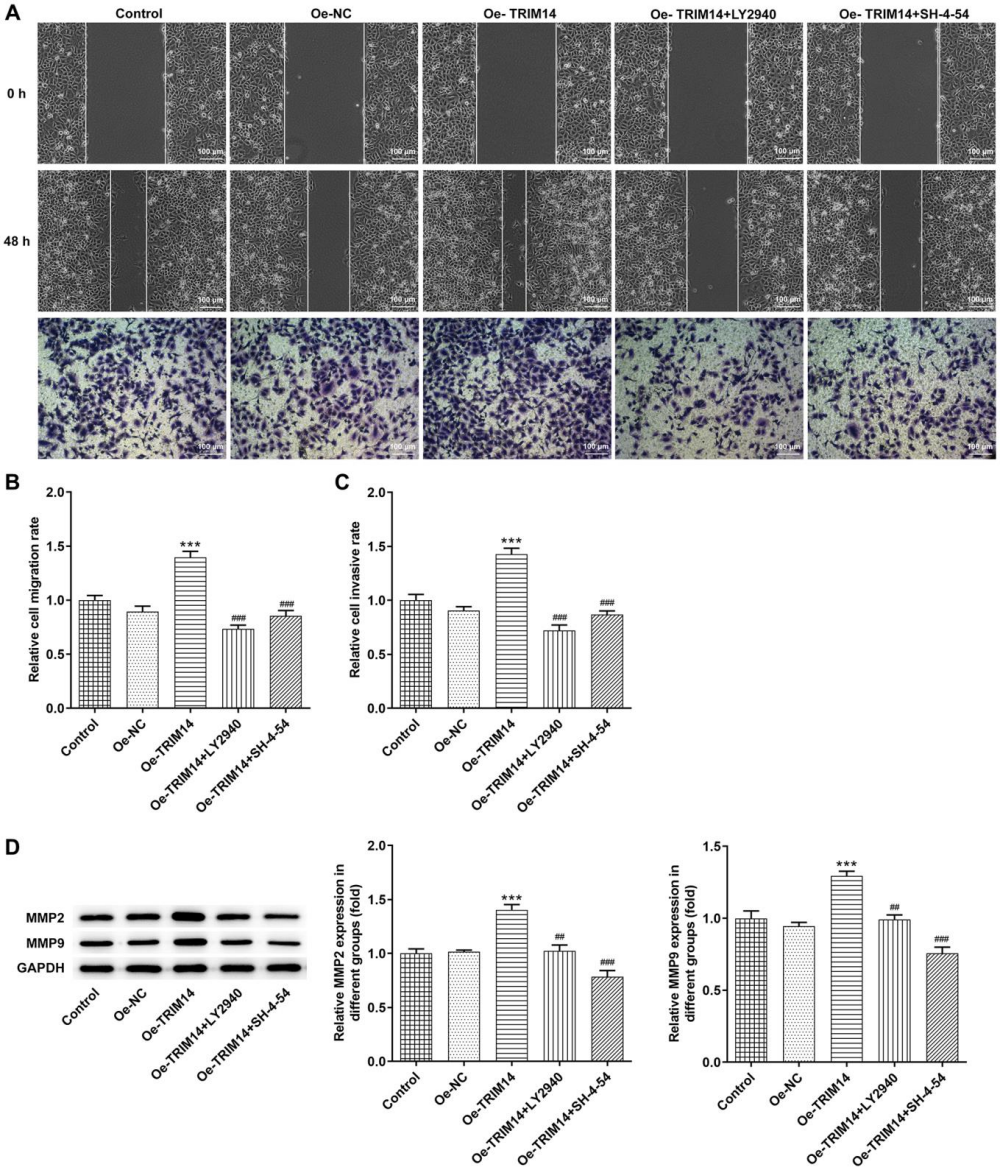
**TRIM14 overexpression enhanced melanoma cell migration and invasion through AKT and STAT3 pathways.** After indicated treatment, the migration and invasion abilities of A375 cells were determined using wound-healing assay and Transwell assay, respectively (**A**–**C**). The protein expression of MMP2 and MMP9 was determined using western blot (**D**). Magnification ×100. ^***^p < 0.001 vs Oe-NC; ^##^, ^###^*p* < 0.01, 0.001 vs Oe-TRIM14.

Then, wound healing and transwell assays were performed to explore impacts of overexpression of TRIM14 on the migration and invasion of melanoma cells. Our further wound healing assay showed that TRIM14 overexpression promoted the migration of melanoma cells, while inhibition of AKT or STAT3 pathway with specific inhibitors partially abolished the migration of melanoma cells induced by TRIM14 overexpression. Overexpression of TRIM14 also increased the number of invasive melanoma cells while blocking AKT or STAT3 pathway did the opposite effects (Figure 8A–8C). Furthermore, levels of MMP2 and MMP9 proteins presented similar results (Figure 8D). These results suggested that AKT and STAT3 participated in modulating melanoma cell migration and invasion that TRIM14 mediated.

### Overexpression of TRIM14 increased melanin synthesis of melanoma cells through AKT and STAT3 pathways

**Figure 9:**
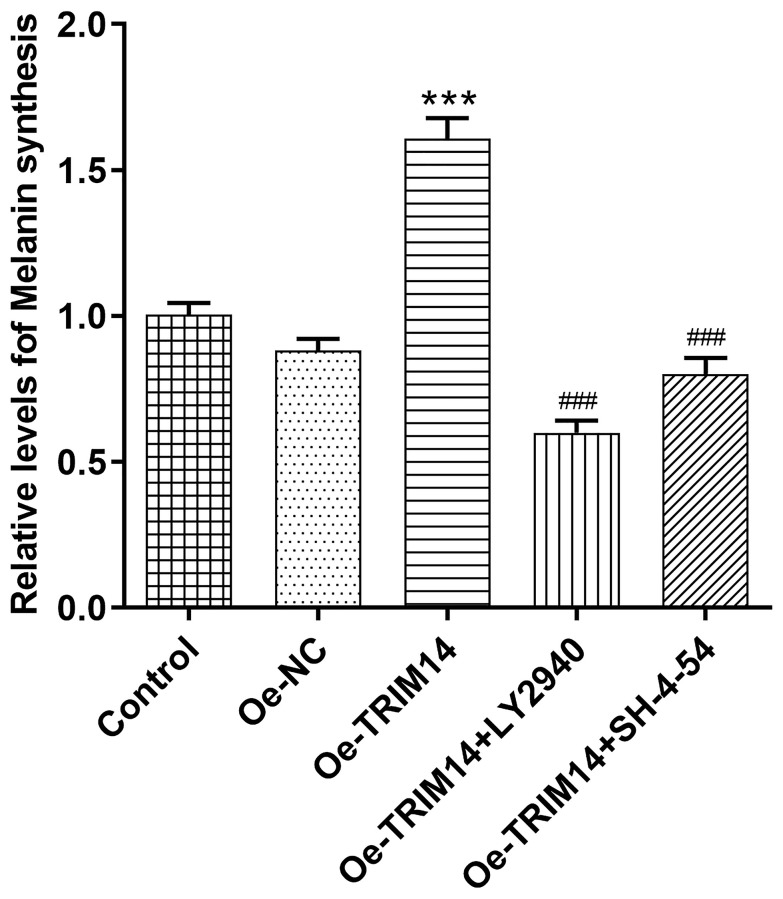
**Overexpression of TRIM14 upregulated melanin synthesis of melanoma cell via AKT and STAT3 pathways.** After indicated treatment, the melanin synthesis abilities of different groups in A375 cells were measured. ^***^p < 0.001 vs Oe-NC; ^###^*p* < 0.001 vs Oe-TRIM14.

After confirming AKT and STAT3 pathways were associated with TRIM14-induced melanoma malignancy, we then further explored whether AKT or STAT3 pathway participated in regulating melanin synthesis of melanoma A375 cells mediated by TRIM14. As shown in Figure 9, compared with control or negative control, TRIM14 overexpression increased levels of melanin synthesis of melanoma cells. However, when AKT or STAT3 pathway was blocked, levels of melanin synthesis of melanoma cells mediated by TRIM14 overexpression were decreased. These results demonstrated that TRIM14 overexpression promoted the synthesis of melanoma cells via AKT and STAT3 pathways.

### TRIM14 overexpression promoted the epithelial-mesenchymal transition of melanoma cells by regulating AKT and STAT3 pathways

**Figure 10:**
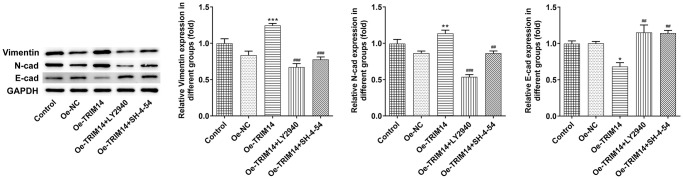
**TRIM14 overexpression induced melanoma cell epithelial-mesenchymal transition through PI3K/AKT and STAT3 pathways.** After indicated treatment, the protein expression of vimentin, N-cadherin, and E-cadherin was measured using western blot. ^*^, ^**^, ^***^p < 0.05, 0.01, 0.001 vs Oe-NC; ^##^, ^###^*p* < 0.01, 0.001 vs Oe-TRIM14.

During tumor cell development, epithelial-mesenchymal transition played an important role [21]. To know the biological function of TRIM14 in the epithelial-mesenchymal transition of A375 cells, melanoma cells were pretreated with AKT or STAT3 specific inhibitors and then transfected with TRIM14 overexpression plasmid. Vimentin, N-cadherin, and E-cadherin were the indicators of epithelial-mesenchymal transition. As shown in Figure 10, overexpression of TRIM14 upregulated levels of epithelial-mesenchymal transition inducers vimentin and N-cadherin and downregulated epithelial-mesenchymal transition inhibitor E-cadherin expression. Moreover, blocking AKT or STAT3 pathway could partially reverse the epithelial-mesenchymal transition induced by TRIM14. These results showed that overexpression of TRIM14 induced epithelial-mesenchymal transition of melanoma cells via AKT and STAT3 pathways.

### TRIM14 regulated melanoma tumor growth through AKT and STAT3 pathways *in vivo*

**Figure 11:**
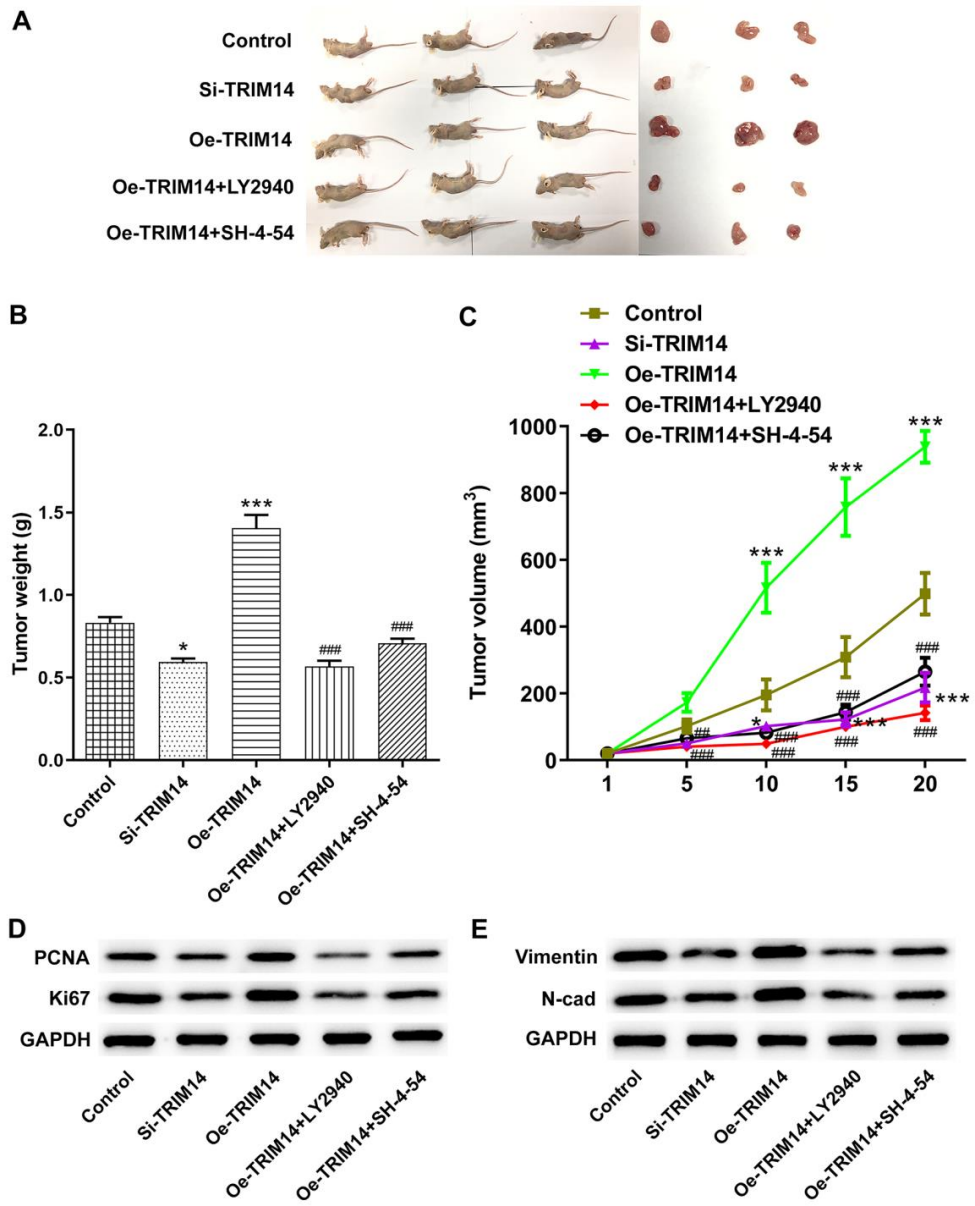
**TRIM14 regulated melanoma tumorigenesis *in vivo*.** A375 melanoma cells were transfected with si-TRIM14 or Oe-TRIM14 plasmid with or without pretreatment of LY2940/SH-4-54. After that, the above cells were injected into nude mice. The tumor was peeled off and weighed on day 20 to weigh and take pictures (**A**–**B**). The tumor growth curve for the animal experiment of each group was recorded and calculated (**C**). The tumor tissues were collected and applied to measure the protein expression of PCNA, Ki67, vimentin, and N-cadherin using western blot (**D**–**E**). ^*^, ^***^p < 0.05, 0.001 vs control; ^###^*p* < 0.001 vs Oe-TRIM14.

Finally, A375 melanoma cells were transfected with si-TRIM14 or Oe-TRIM14 plasmid with or without pretreatment of LY2940/SH-4-54. After that, the above cells were injected into nude mice to explore the roles of TRIM14 *in vivo*. As shown in Figure 11A–11C, knockdown of TRIM14 reduced the size and weight of tumor, while overexpression of TRIM14 promoted melanoma tumor growth. What’s more, inhibition of AKT or STAT3 pathway partially abolished the growth of melanoma induced by TRIM14 overexpression. Proliferating cell nuclear antigen (PCNA), and Ki67 are standard markers of proliferation which are commonly applied to evaluate the growth fraction of cell population [22]. Then, knockdown of TRIM14 exhibited inhibitory effects on the expression of PCAN, Ki67, vimentin and N-cadherin, while overexpression of TRIM14 did the opposite effects, which were partially abolished by inhibition of AKT or STAT3 pathway (Figure 11D, 11E). These results revealed the important roles of TRIM14 in regulating melanoma tumor growth *in vivo*.

## DISCUSSION

Melanoma is one of the lethal tumors in humans, and patients with malignant melanoma generally have a poor overall survival [1]. One of the most common complications of patients with malignant melanoma is metastasis to the brain, and the 5-year survival rate of this type of patients has dropped to about 23% [23]. To date, there are still no effective methods for the treatment of melanoma. In recent decades, targeted therapy and immunotherapy have gradually been considered as promising approaches to melanoma therapy [24]. In this study, we reported the biological roles of the oncogene TRIM14 in melanoma. TRIM14 was upregulated in melanoma cell lines to promote melanoma cell proliferation, clone formation, migration, invasion, and epithelial-mesenchymal transition through PI3K/AKT and STAT3 pathways.

TRIM14, an E3 ubiquitin ligase, belongs to the TRIM family [25]. Previous studies have reported that TRIM14 acted as an oncogene in many cancers and participated in regulating tumor malignancy. Our results showed that TRIM14 was upregulated in melanoma cell lines. Knockdown of TRIM14 suppressed melanoma cell proliferation, migration, invasion, and melanin synthesis of melanoma cells, while TRIM14 overexpression did the opposite. Furthermore, overexpressed TRIM14 also promoted tumor cells epithelial-mesenchymal transition. In gastric cancer, TRIM14 was aberrantly upregulated in cancer tissues and cell lines. Overexpression of TRIM14 promoted gastric cancer cell invasion and epithelial-mesenchymal transition [26]. In glioma, TRIM14 was overexpressed in tumor tissues and cell lines, and TRIM14 levels were negatively correlated with the survival time of glioma patients. TRIM14 promoted epithelial-mesenchymal transition in glioma cells [18]. Furthermore, up-regulation of TRIM14 was found in breast cancer cell lines to promote breast cancer cell proliferation [17]. This research supported our results that TRIM14 played oncogenic roles in melanoma. However, in non-small cell lung cancer, TRIM14 was downregulated and functioned as a tumor suppressor to block tumor cells proliferation [27]. Tumor heterogeneity may account for this phenomenon [28].

Previous studies reported that TRIM14 could colocalize PTEN in the cytoplasm and induce PTEN degradation in an ubiquitination-dependent manner, which in turn activate PI3K/AKT and STAT3 pathways [15, 16, 20]. Our results showed that knockdown of TRIM14 increased levels of PTEN protein. To our delight, AKT and STAT3 pathways were also inhibited following TRIM14 knockdown. These results indicated that AKT and STAT3 pathways might participate in TRIM14 mediated melanoma malignancy. To further verify this hypothesis, we constructed the TRIM14 overexpression plasmid. Our results presented that pretreated melanoma cells with AKT or STAT3 pathway-specific inhibitor could partially reverse TRIM14 induced tumor cell proliferation, clone formation capacity, invasion, epithelial-mesenchymal transition as well as melanin synthesis capacity.

AKT pathway is a key mediator in cancer, and once activated, AKT promotes cellular proliferation, invasion, and angiogenesis, etc by regulating the function of many downstream related proteins [29]. Apart from TRIM14, the AKT pathway was also activated by other TRIM family members. For instance, TRIM27 enhanced esophageal squamous cell carcinoma growth via activation of the AKT pathway [30]. In breast cancer, TRIM47 knockdown suppressed tumorigenesis and progression through inhibition of ATK pathway [31]. Moreover, it’s reported that TRIM31 regulated gallbladder cancer cell proliferation and invasion by suppressing ATK pathway. These results demonstrate that the TRIM family may function their roles via a similar manner.

STAT3 pathway was found to be hyperactivated in many cancers, and aberrant activation generally means a poor clinical prognosis [32]. In our study, we reported that STAT3 pathway activation was involved in melanoma malignancy mediated by TRIM14. The STAT3 pathway has been shown to participate in the promotion of colorectal cancer cell invasion induced by TRIM14 [16]. Previously, Hu et al. found that TRIM14 promoted breast cancer cell proliferation and inhibited cell apoptosis by targeting the SPHK1/STAT3 pathway [17]. Furthermore, TRIM14 promoted papillary thyroid carcinoma cell proliferation via the interaction with SOCS1, a negative regulator of the STAT3 activation [33]. The largest difference between the previous finding and ours was that our results revealed TRIM14 might mediate PTEN degradation to activate STAT3 pathway in melanoma. Nevertheless, the exact mechanism of how TRIM14 induces PTEN degradation needs further exploration.

In summary, our study found that TRIM14 was abnormally overexpressed in melanoma cell lines. Knockdown of TRIM14 suppressed melanoma cell proliferation, invasion, and epithelial-mesenchymal transition through PI3K/AKT and STAT3 pathways. Inhibition of PI3K/AKT or STAT3 pathway partially abolished the promotion of melanoma malignancy induced by TRIM14 overexpression. *In vivo* assay also supported the above findings. Our study may provide some suggestions for the treatment of melanoma.

## References

[1] Webster MR, Fane ME, Alicea GM, Basu S, Kossenkov AV, Marino GE, Douglass SM, Kaur A, Ecker BL, Gnanapradeepan K, Ndoye A, Kugel C, Valiga A, et al. Paradoxical Role for Wild-Type p53 in Driving Therapy Resistance in Melanoma. Mol Cell. 2020; 77:633–44.e5. 10.1016/j.molcel.2019.11.009 31836388 PMC7419227

[2] Lin WM, Fisher DE. Signaling and Immune Regulation in Melanoma Development and Responses to Therapy. Annu Rev Pathol. 2017; 12:75–102. 10.1146/annurev-pathol-052016-100208 27959628

[3] Queirolo P, Boutros A, Tanda E, Spagnolo F, Quaglino P. Immune-checkpoint inhibitors for the treatment of metastatic melanoma: a model of cancer immunotherapy. Semin Cancer Biol. 2019; 59:290–97. 10.1016/j.semcancer.2019.08.001 31430555

[4] Bai X, Fisher DE, Flaherty KT. Cell-state dynamics and therapeutic resistance in melanoma from the perspective of MITF and IFNγ pathways. Nat Rev Clin Oncol. 2019; 16:549–62. 10.1038/s41571-019-0204-6 30967646 PMC7185899

[5] da Silveira Nogueira Lima JP, Georgieva M, Haaland B, de Lima Lopes G. A systematic review and network meta-analysis of immunotherapy and targeted therapy for advanced melanoma. Cancer Med. 2017; 6:1143–53. 10.1002/cam4.1001 28463413 PMC5463084

[6] Williams FP, Haubrich K, Perez-Borrajero C, Hennig J. Emerging RNA-binding roles in the TRIM family of ubiquitin ligases. Biol Chem. 2019; 400:1443–64. 10.1515/hsz-2019-0158 31120853

[7] Zhang R, Li SW, Liu L, Yang J, Huang G, Sang Y. TRIM11 facilitates chemoresistance in nasopharyngeal carcinoma by activating the β-catenin/ABCC9 axis via p62-selective autophagic degradation of Daple. Oncogenesis. 2020; 9:45. 10.1038/s41389-020-0229-9 32382014 PMC7206012

[8] Han Y, Tan Y, Zhao Y, Zhang Y, He X, Yu L, Jiang H, Lu H, Tian H. TRIM23 overexpression is a poor prognostic factor and contributes to carcinogenesis in colorectal cancer. J Cell Mol Med. 2020; 24:5491–500. 10.1111/jcmm.15203 32227572 PMC7214184

[9] Zhao Z, Wang Y, Yun D, Huang Q, Meng D, Li Q, Zhang P, Wang C, Chen H, Lu D. TRIM21 overexpression promotes tumor progression by regulating cell proliferation, cell migration and cell senescence in human glioma. Am J Cancer Res. 2020; 10:114–30. 32064156 PMC7017742

[10] Liu Y, Tao S, Liao L, Li Y, Li H, Li Z, Lin L, Wan X, Yang X, Chen L. TRIM25 promotes the cell survival and growth of hepatocellular carcinoma through targeting Keap1-Nrf2 pathway. Nat Commun. 2020; 11:348. 10.1038/s41467-019-14190-2 31953436 PMC6969153

[11] Yeh I, Botton T, Talevich E, Shain AH, Sparatta AJ, de la Fouchardiere A, Mully TW, North JP, Garrido MC, Gagnon A, Vemula SS, McCalmont TH, LeBoit PE, Bastian BC. Activating MET kinase rearrangements in melanoma and Spitz tumours. Nat Commun. 2015; 6:7174. 10.1038/ncomms8174 26013381 PMC4446791

[12] Sutton SK, Cheung BB, Massudi H, Tan O, Koach J, Mayoh C, Carter DR, Marshall GM. Heterozygous loss of keratinocyte TRIM16 expression increases melanocytic cell lesions and lymph node metastasis. J Cancer Res Clin Oncol. 2019; 145:2241–50. 10.1007/s00432-019-02981-5 31342168 PMC6708510

[13] Hutchinson KE, Lipson D, Stephens PJ, Otto G, Lehmann BD, Lyle PL, Vnencak-Jones CL, Ross JS, Pietenpol JA, Sosman JA, Puzanov I, Miller VA, Pao W. BRAF fusions define a distinct molecular subset of melanomas with potential sensitivity to MEK inhibition. Clin Cancer Res. 2013; 19:6696–702. 10.1158/1078-0432.CCR-13-1746 24345920 PMC3880773

[14] Lionnard L, Duc P, Brennan MS, Kueh AJ, Pal M, Guardia F, Mojsa B, Damiano MA, Mora S, Lassot I, Ravichandran R, Cochet C, Aouacheria A, et al. TRIM17 and TRIM28 antagonistically regulate the ubiquitination and anti-apoptotic activity of BCL2A1. Cell Death Differ. 2019; 26:902–17. 10.1038/s41418-018-0169-5 30042493 PMC6461866

[15] Xu G, Guo Y, Xu D, Wang Y, Shen Y, Wang F, Lv Y, Song F, Jiang D, Zhang Y, Lou Y, Meng Y, Yang Y, Kang Y. TRIM14 regulates cell proliferation and invasion in osteosarcoma via promotion of the AKT signaling pathway. Sci Rep. 2017; 7:42411. 10.1038/srep42411 28205534 PMC5311867

[16] Jin Z, Li H, Hong X, Ying G, Lu X, Zhuang L, Wu S. TRIM14 promotes colorectal cancer cell migration and invasion through the SPHK1/STAT3 pathway. Cancer Cell Int. 2018; 18:202. 10.1186/s12935-018-0701-1 30555277 PMC6288942

[17] Hu G, Pen W, Wang M. TRIM14 Promotes Breast Cancer Cell Proliferation by Inhibiting Apoptosis. Oncol Res. 2019; 27:439–47. 10.3727/096504018X15214994641786 29562956 PMC7848417

[18] Tan Z, Song L, Wu W, Zhou Y, Zhu J, Wu G, Cao L, Song J, Li J, Zhang W. TRIM14 promotes chemoresistance in gliomas by activating Wnt/β-catenin signaling via stabilizing Dvl2. Oncogene. 2018; 37:5403–15. 10.1038/s41388-018-0344-7 29867201

[19] Feng S, Cai X, Li Y, Jian X, Zhang L, Li B. Tripartite motif-containing 14 (TRIM14) promotes epithelial-mesenchymal transition via ZEB2 in glioblastoma cells. J Exp Clin Cancer Res. 2019; 38:57. 10.1186/s13046-019-1070-x 30728039 PMC6364431

[20] Shen W, Jin Z, Tong X, Wang H, Zhuang L, Lu X, Wu S. TRIM14 promotes cell proliferation and inhibits apoptosis by suppressing PTEN in colorectal cancer. Cancer Manag Res. 2019; 11:5725–35. 10.2147/CMAR.S210782 31296997 PMC6598940

[21] Campbell K, Lebreton G, Franch-Marro X, Casanova J. Differential roles of the Drosophila EMT-inducing transcription factors Snail and Serpent in driving primary tumour growth. PLoS Genet. 2018; 14:e1007167. 10.1371/journal.pgen.1007167 29420531 PMC5821384

[22] Juríková M, Danihel Ľ, Polák Š, Varga I. Ki67, PCNA, and MCM proteins: Markers of proliferation in the diagnosis of breast cancer. Acta Histochem. 2016; 118:544–52. 10.1016/j.acthis.2016.05.002 27246286

[23] Schmidberger H, Rapp M, Ebersberger A, Hey-Koch S, Loquai C, Grabbe S, Mayer A. Long-term survival of patients after ipilimumab and hypofractionated brain radiotherapy for brain metastases of malignant melanoma: sequence matters. Strahlenther Onkol. 2018; 194:1144–51. 10.1007/s00066-018-1356-5 30298365 PMC6267133

[24] Mancuso P, Tricarico R, Bhattacharjee V, Cosentino L, Kadariya Y, Jelinek J, Nicolas E, Einarson M, Beeharry N, Devarajan K, Katz RA, Dorjsuren DG, Sun H, et al. Thymine DNA glycosylase as a novel target for melanoma. Oncogene. 2019; 38:3710–28. 10.1038/s41388-018-0640-2 30674989 PMC6563616

[25] Cui J, Xu X, Li Y, Hu X, Xie Y, Tan J, Qiao W. TRIM14 expression is regulated by IRF-1 and IRF-2. FEBS Open Bio. 2019; 9:1413–20. 10.1002/2211-5463.12682 31150153 PMC6668374

[26] Wang F, Ruan L, Yang J, Zhao Q, Wei W. TRIM14 promotes the migration and invasion of gastric cancer by regulating epithelial-to-mesenchymal transition via activation of AKT signaling regulated by miR-195-5p. Oncol Rep. 2018; 40:3273–84. 10.3892/or.2018.6750 30272351 PMC6196628

[27] Hai J, Zhu CQ, Wang T, Organ SL, Shepherd FA, Tsao MS. TRIM14 is a Putative Tumor Suppressor and Regulator of Innate Immune Response in Non-Small Cell Lung Cancer. Sci Rep. 2017; 7:39692. 10.1038/srep39692 28059079 PMC5216374

[28] Roma-Rodrigues C, Mendes R, Baptista PV, Fernandes AR. Targeting Tumor Microenvironment for Cancer Therapy. Int J Mol Sci. 2019; 20:840. 10.3390/ijms20040840 30781344 PMC6413095

[29] Revathidevi S, Munirajan AK. Akt in cancer: Mediator and more. Semin Cancer Biol. 2019; 59:80–91. 10.1016/j.semcancer.2019.06.002 31173856

[30] Ma L, Yao N, Chen P, Zhuang Z. TRIM27 promotes the development of esophagus cancer via regulating PTEN/AKT signaling pathway. Cancer Cell Int. 2019; 19:283. 10.1186/s12935-019-0998-4 31719796 PMC6839104

[31] Wang Y, Liu C, Xie Z, Lu H. Knockdown of TRIM47 inhibits breast cancer tumorigenesis and progression through the inactivation of PI3K/Akt pathway. Chem Biol Interact. 2020; 317:108960. 10.1016/j.cbi.2020.108960 31981573

[32] Johnson DE, O’Keefe RA, Grandis JR. Targeting the IL-6/JAK/STAT3 signalling axis in cancer. Nat Rev Clin Oncol. 2018; 15:234–48. 10.1038/nrclinonc.2018.8 29405201 PMC5858971

[33] Sun W, Wang Y, Li D, Wu Y, Ji Q, Sun T. Tripartite motif containing 14: An oncogene in papillary thyroid carcinoma. Biochem Biophys Res Commun. 2020; 521:360–67. 10.1016/j.bbrc.2019.10.127 31668806

